# Simulating Multilevel Dynamics of Antimicrobial Resistance in a Membrane Computing Model

**DOI:** 10.1128/mBio.02460-18

**Published:** 2019-01-29

**Authors:** Marcelino Campos, Rafael Capilla, Fernando Naya, Ricardo Futami, Teresa Coque, Andrés Moya, Val Fernandez-Lanza, Rafael Cantón, José M. Sempere, Carlos Llorens, Fernando Baquero

**Affiliations:** a Department of Microbiology, Ramón y Cajal University Hospital, IRYCIS, Madrid, Spain; b Biotechvana, Paterna, Valencia, Spain; c Department of Information Systems and Computation (DSIC), Universitat Politècnica de València, Valencia, Spain; d Antibiotic Resistance and Bacterial Virulence Unit (HRYC-CSIC), Superior Council of Scientific Research (CSIC), Madrid, Spain; e Network Research Center for Epidemiology and Public Health (CIBER-ESP), Madrid, Spain; f Integrative Systems Biology Institute, University of Valencia and Spanish Research Council (CSIC), Paterna, Valencia, Spain; g Foundation for the Promotion of Sanitary and Biomedical Research in the Valencian Community (FISABIO), Valencia, Spain; h Bioinformatics Support Unit, IRYCIS, Madrid, Spain; Indiana University Bloomington; Harvard School of Public Health; Former Professor of Medicine, Case Western Reserve University; University of Seville

**Keywords:** antibiotic resistance, membrane computing, multilevel, computer modeling, mathematical modeling

## Abstract

Membrane computing is a bio-inspired computing paradigm whose devices are the so-called membrane systems or P systems. The P system designed in this work reproduces complex biological landscapes in the computer world. It uses nested “membrane-surrounded entities” able to divide, propagate, and die; to be transferred into other membranes; to exchange informative material according to flexible rules; and to mutate and be selected by external agents. This allows the exploration of hierarchical interactive dynamics resulting from the probabilistic interaction of genes (phenotypes), clones, species, hosts, environments, and antibiotic challenges. Our model facilitates analysis of several aspects of the rules that govern the multilevel evolutionary biology of antibiotic resistance. We examined a number of selected landscapes where we predict the effects of different rates of patient flow from hospital to the community and vice versa, the cross-transmission rates between patients with bacterial propagules of different sizes, the proportion of patients treated with antibiotics, and the antibiotics and dosing found in the opening spaces in the microbiota where resistant phenotypes multiply. We also evaluated the selective strengths of some drugs and the influence of the time 0 resistance composition of the species and bacterial clones in the evolution of resistance phenotypes. In summary, we provide case studies analyzing the hierarchical dynamics of antibiotic resistance using a novel computing model with reciprocity within and between levels of biological organization, a type of approach that may be expanded in the multilevel analysis of complex microbial landscapes.

## Abstract (precis)

The work that we present here represents the culmination of many years of investigation in looking for a suitable methodology to simulate the multihierarchical processes involved in antibiotic resistance. Everything started with our early appreciation of the different independent but embedded biological units that shape the biology, ecology, and evolution of antibiotic-resistant microorganisms. Genes, plasmids carrying these genes, cells hosting plasmids, populations of cells, microbial communities, and host's populations constitute a complex system where changes in one component might influence the other ones. How would it be possible to simulate such a complexity of antibiotic resistance as it occurs in the real world? Can the process be predicted, at least at the local level? A few years ago, and because of their structural resemblance to biological systems, we realized that membrane computing procedures could provide a suitable frame to approach these questions. Our manuscript describes the first application of this modeling methodology to the field of antibiotic resistance and offers a bunch of examples—just a limited number of them in comparison with the possible ones to illustrate its unprecedented explanatory power.

## INTRODUCTION

Antibiotic (Ab) resistance is the result of the complex interaction of discrete evolutionary entities placed in different hierarchical levels of biological organization, including resistance genes, mobile genetic elements, clones, species, genetic exchange communities, microbiomes, and the hosts of these bacterial ensembles placed in particular biological environments (1–3). Under the influence of external environmental variations (such as exposure to antibiotics), all of these evolutionary entities might have independent rates of variation and selection, but as they are hierarchically linked, the changes in each of them can influence all of the other entities (4), as they constitute a global “nested biological system” (5).

Membrane computing is an individual-based natural computing paradigm aimed at abstract computing ideas and models from the structure and the functioning of living cells, as well as from the way that the cells are organized in tissues or higher-order structures (6, 7). Included among the computational models using this paradigm are “P systems,” consisting in placing objects (in our case, biological entities) into virtual cell-like or tissue-like membrane structures such that one membrane or one cell, respectively, represents a hierarchical level, that is, a region of the embedded system. For instance, each bacterial cell is a membrane containing plasmids (as objects), and a plasmid is a membrane containing genes (as objects). The mobility of entities, objects, across membranes is possible according to preestablished rewriting rules, and the collection of multisets of entities evolves in synchronous, parallel, and nondeterministic manners. The objects have assigned rules with respect to passing through membranes (to mimic intracellular or intercellular transmission) (8, 9), to dissolving (to mimic elimination), and to dividing themselves (to mimic replication). In this work, we used a P system to simulate multilevel dynamics of antibiotic resistance, based on our first published prototype (8, 9). This computational model facilitates an approach that is computationally hard to accomplish or simply impossible to address experimentally. Our work allows the estimation and evaluation of global and specific effects on the frequency of each of the biological entities involved in antibiotic resistance occurring because of changes taking place (as following antibiotic exposure) in one or (simultaneously) in several of them. Note that, albeit antibiotic resistance is a major problem in public health, in terms of biosystems, it is only a particular example of “evolution in action.” Our model can be easily applied to many other complex evolutionary landscapes, involving other genes, phenotypes, cells, populations, communities, and ecosystems.

## RESULTS

The main objective of the present work is to present the possibilities of membrane computational modeling as a powerful tool in the evaluation of the factors that, at various biological levels, might influence the dynamics of antibiotic resistance. The results provided below should not be taken as predictions of the evolution of resistance but instead as illustrations of some of the possibilities of this model for the study of the multilevel dynamics of resistance, by simultaneously changing parameters in state variables and observing after a single run the effect on the frequency of resistant species and populations. Note that the model is probabilistic and that the rules are selected in a probabilistic way. So, each computation produces an output such that the results obtained are not entirely identical in consecutive runs of the program but are relatively close (see Fig. S1 in the supplemental material). In the following paragraphs, antibiotics (Ab) are named AbA, AbC, and AbF and the corresponding resistances (R) AbAR, AbCR, and AbFR, respectively; to facilitate reading, we suggest the identification of AbA as the aminopenicillins, AbC as cefotaxime-ceftazidime, and AbF as fluoroquinolones (FLQs), using the initials of three of the major groups of antibiotics used in clinical practice (Table 1).

**TABLE 1:** Abbreviations used in the text and figures

Abbreviation(s)	Meaning
Bacterial populations in the model	
Ec	Escherichia coli
Kp	Klebsiella pneumoniae
Ef	Enterococcus faecium
Pa	Pseudomonas aeruginosa

Antibiotics (Ab) and resistance phenotypes (R)	
AbA, AbAR	Antibiotic A (aminopenicillins)
AbC, AbCR	Antibiotic C (cefotaxime)
AbF, AbFR	Antibiotic F (fluoroquinolones)

E. coli and K. pneumoniae resistance phenotypes (in figures)	
Ec0, Kp0	Susceptible
EcA, KpA	Resistant to antibiotic A
EcC, KpC	Resistant to antibiotic C
EcF, KpF	Resistant to antibiotic F
EcAC, KpAC	Resistant to antibiotics A and C
EcAF, KPAF	Resistant to antibiotics A and F
EcACF, KpACF	Resistant to antibiotics A, C, and F

E. coli starting clones	
Ecc0	Antibiotic susceptible
EccA	Resistant to antibiotic A
EccF	Resistant to antibiotic F
EccAF	Resistant to antibiotics A and F

*Enterococcus* resistance phenotypes	
Ef(1)0	Antibiotic A susceptible
Ef1(1)A	Resistant to antibiotic A
Ef(2)AF	Resistant to antibiotics A and F

Conjugative elements	
PL1	Plasmid 1
CO1	Conjugative element in *Enterococcus*

10.1128/mBio.02460-18.1 FIG S1 Three consecutive model iterations, in the three panels of the figure, representing the dynamics of E. coli resistance phenotypes in the hospital compartment. As the model includes several stochastic and probabilistic steps, the results obtained in replicated runs of the program are not entirely identical. However, they are extremely close. Download FIG S1, EPS file, 2.5 MB. Copyright © 2019 Campos et al. 2019 Campos et al. This content is distributed under the terms of the Creative Commons Attribution 4.0 International license.

### The basic scenario in the hospital and community compartments. (i) Dynamics of bacterial resistance phenotypes in Escherichia coli.

**FIG 1:**
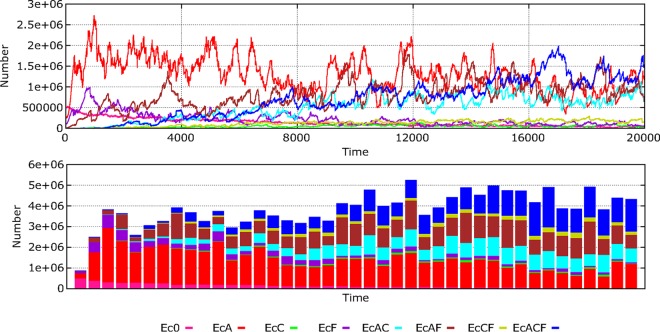
Dynamics of bacterial resistance phenotypes in E. coli. Pink, susceptible; red, AbAR (AMP); violet, AbFR (FLQ); brown, AbAR and AbFR; light blue, AbAR and AbCR; dark blue, AbAR, AbCR, and AbFR; light green, AbCR; olive green, AbCR and AbFR. In ordinates, numbers of hecto-cells (h-cells; packages of 100 identical cells) in all hosts per milliliter (with each host represented by 1 ml of colonic content); in abscissa, time (1,000 steps, roughly equivalent to 42 days).

**FIG 2:**
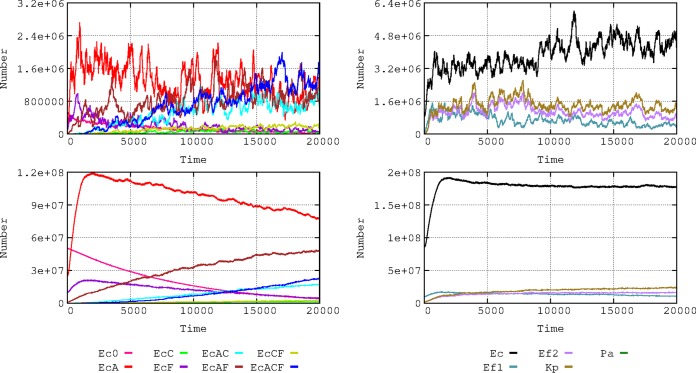
Comparative dynamics of E. coli phenotypes in the hospital (up, left) and the community (down, left). In the right part, species dynamics in the hospital (up) and the community (down): E. coli (black), K. pneumoniae (yellow green), E. faecium AbAS (dark green), and E. faecium AbAR (violet). P. aeruginosa is not visible in this representation (low numbers).

Waves of successive replacements of resistance phenotypes in hospital-based E. coli strains during 20,000 time steps (about 2.3 years, as the time steps represent approximately 1 h/step) are illustrated in Fig. 1. The main features of this process, mimicking clonal interference, are as follows: (i) a sharp decrease in the density of the fully susceptible phenotype (pink line); (ii) a rapid increase of the phenotype AbAR (aminopenicillin resistance), resulting from the transfer of the plasmid with AbAR to the susceptible population and consequent selection (red); (iii) increase by selection and, marginally, by acquisition of mutational resistance of the phenotype AbFR (fluoroquinolone resistance) (violet); (iv) increase of double resistances AbAR and AbFR by acquisition of an AbFR mutation with the organisms of AbAR-only phenotype and by the transfer of the plasmid encoding AbAR from the AbAR-only phenotype to the AbFR-only phenotype (brown); (v) increase of the phenotype with double resistances AbAR and AbCR by capture by the AbAR-only predominant phenotype of a plasmid containing AbCR (cefotaxime resistance) that originated in Klebsiella pneumoniae (light blue); (vi) almost simultaneous emergence but later predominance of the multiresistant organisms with phenotype AbAR, AbCR, and AbFR by mutational acquisition of AbFR by the doubly resistant phenotype AbAR-AbCR and, also, of the plasmid-mediated AbCR by the AbAR-AbFR phenotype (dark blue); (vii) close in time, emergence (but with low density) of the phenotype AbCR-only by the acquisition of the plasmid encoding AbCR by the fully susceptible phenotype and the AbAR phenotype and loss of plasmid-mediated AbAR by incompatibility with the incoming plasmid (light green); and (viii) the acquisition of the AbFR mutation by the AbCR-only phenotype or by plasmid reception of an AbCR trait from K. pneumoniae in AbFR, giving rise to the phenotype AbCR-AbFR (olive green). In the community, where the antibiotic exposure is less frequent, a similar dynamic sequence occurs but at a much lower rate (Fig. 2).

### (ii) Dynamics of bacterial species.

Antibiotic use and antibiotic resistance influence the long-term dynamics of bacterial species in the hospital environment (Fig. 2C and D). Under the conditions of our basic scenario, E. coli populations (black) tend to prevail. Enterococcus faecium (violet) and K. pneumoniae (yellow-green) populations were maintained during the experiment. In the community, E. coli has a stronger dominance over other species, and similar dynamics occur as in the hospital, at lower rates.

Klebsiella pneumoniae (Fig. S3) is intrinsically resistant to AbA, and in our case it harbors a plasmid encoding AbCR (cefotaxime [CTX]) and a mutation encoding AbFR (fluoroquinolone [FLQ]). In the hospital, the AbCR phenotype is readily selected. However, because of the high density of E. coli populations with the plasmid-mediated AbAR, several *Klebsiella* strains receive this plasmid. These *Klebsiella* strains receive no benefit from this plasmid because they are intrinsically aminopenicillin resistant, but incompatibility with the plasmid determining AbCR occurs, eliminating AbCR from the recipients and giving rise to the phenotype AbAR-AbFR (purple). That contributes to the decline in AbCR-containing phenotypes (olive green). In any case, the dominance of E. coli prevents significant growth of K. pneumoniae. Enterococcus faecium (Fig. S3) is intrinsically resistant to AbC (AbCR, CTX), but there are two variants, one AbA (aminopenicillin [AMP]) susceptible and the other AbA resistant, the latter of which has AbFR also. However, the AbAS variant can acquire the AbAR trait from the resistant one by (infrequent) horizontal genetic transfer and can become an AbAR donor. There is replacement dynamics of AbAS by the AbAR phenotype.

### (iii) Influence of baseline resistance composition on the dynamics of bacterial species.

**FIG 3:**
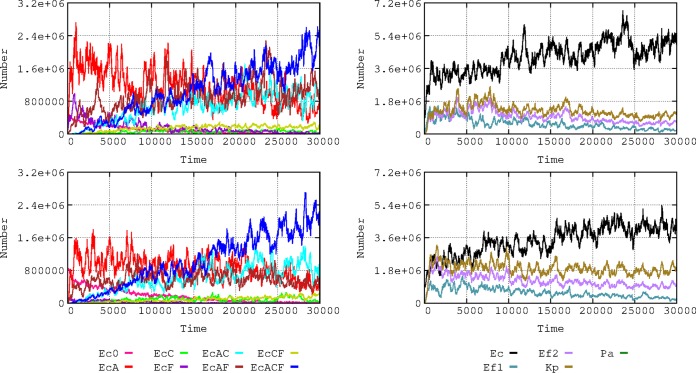
Influence of baseline E. coli resistance phenotype composition on the dynamics of bacterial species. On the left, data represent comparative dynamics of E. coli phenotypes in the basic hospital scenario (top) and with reduced numbers of resistant phenotypes (bottom). Colors and axes are as described for Fig. 1. On the right, data represent comparative dynamics of bacterial species in the basic model (top) and the reduced basal resistances (bottom); colors are as described for Fig. 2.

The local evolution of antibiotic resistance can depend on the baseline composition of susceptible and resistant bacterial populations (Fig. 3). In a baseline scenario, we consider a density of 8,600 h-cells (1 h-cell = 100 identical cells; see the section “Quantitative structure of the basic model application” below) of E. coli among which 5,000 cells are susceptible, 2,500 have plasmid-mediated aminopenicillin resistance (PL1-AbAR), 1,000 have fluoroquinolone resistance (AbFR), and 100 combine both resistances. To mimic a “more-susceptible scenario,” values were changed to 8,000 susceptible cells, 500 with PL1-AbAR, 50 with AbFR, and 50 with PL1-AbAR and AbFR. Higher proportions of susceptible E. coli cells facilitate the increase in the populations of the more resistant organisms, K. pneumoniae and AbAR E. faecium. Because of the selection of K. pneumoniae (olive green) harboring cefotaxime resistance (PL1-AbCR) and because of the ability of transfer of the PL1 plasmid to E. coli, the proportion of E. coli cells with cefotaxime resistance (mainly light and dark blue) increases in the scenario with a lower resistance baseline for E. coli. This example illustrates the hypothesis that a higher prevalence of resistance in the E. coli component of the gut flora might reduce the frequency of other resistant organisms, which might inspire interventions directed to restore the susceptibility in particular species (10, 11).

### (iv) Single-clone E. coli dynamics: influence of baseline resistances.

**FIG 4:**
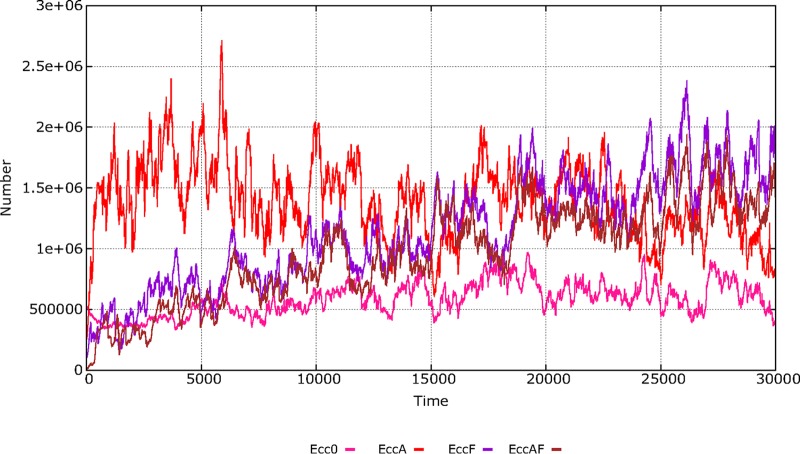
Single-clone E. coli dynamics in the hospital: influence of baseline resistances. In pink, clone Ecc0 starting with full susceptibility; in red, with AbAR (EccA); in violet, with AbFR (EccF); in brown, with AbAR and AbFR (EccAF).

In the previous analysis, subpopulations of E. coli were characterized by their antibiotic resistance phenotype (phenotype populations). Alternatively, we can follow the evolution of four independent E. coli clones, each tagged in the model with particular signals (unrelated with AbR), namely, E. coli clone 0 (Ecc0), EccA, EccF, and EccAF (see Table 1), and, starting with specific resistance traits, allowing for the possibility that the frequency of these “ancestor clones” within a clone might change through time by the gain or loss of a trait. Fig. 4 shows the densities of these ancestor clones through time. The details of the sequential trait acquisitions for each of these clones are shown in Fig. S2. The fully susceptible E. coli clone (Ecc0) first acquires AbAR (red) and AbCR (green). The AbAR phenotype facilitates the capture by lateral gene transfer of AbCR (CTX), giving rise to the double AbAR-AbCR phenotype (light blue). The incorporation of AbF-R (violet; FLQ) in the fully susceptible clone occurs early (later in the AbAR population) such that the rise of the multiresistant phenotype (dark blue) occurs later and again at low numbers. The presence of the AbAR trait in the clone at time zero (EccA) increases the success of the clone and includes the acquisition of AbFR and the multiresistant phenotype. Interestingly, the presence of AbFR (fluoroquinolone resistance) at the origin (EccF) was critical for the enhancement of the numbers of doubly resistant and multiresistant phenotypes. The clones that were more susceptible at the origin remain relatively stable in numbers, suggesting that clonal composition tends to level off along the continued challenges under antibiotic exposure.

10.1128/mBio.02460-18.2 FIG S2 Dynamics of E. coli clones starting with different resistance phenotypes in the hospital compartment. On the left half, from the top down, Ecc0 starting without resistance, EccA starting with AbAR, EccF starting with AbFR, and EccAF with AbAR and AbFR. On the right half of the figure, the same in logarithmic representation, allowing to minority phenotypes to be revealed. Download FIG S2, EPS file, 2.5 MB. Copyright © 2019 Campos et al. 2019 Campos et al. This content is distributed under the terms of the Creative Commons Attribution 4.0 International license.

10.1128/mBio.02460-18.3 FIG S3 Dynamics of K. pneumoniae (top) and of susceptible E. faecium Ef(1) (middle) and Ef(2) (bottom) AbAR in the hospital and community (left and right columns, respectively). Download FIG S3, EPS file, 1.5 MB. Copyright © 2019 Campos et al. 2019 Campos et al. This content is distributed under the terms of the Creative Commons Attribution 4.0 International license.

### (v) Dynamics of mobile genetic elements and resistance traits.

**FIG 5:**
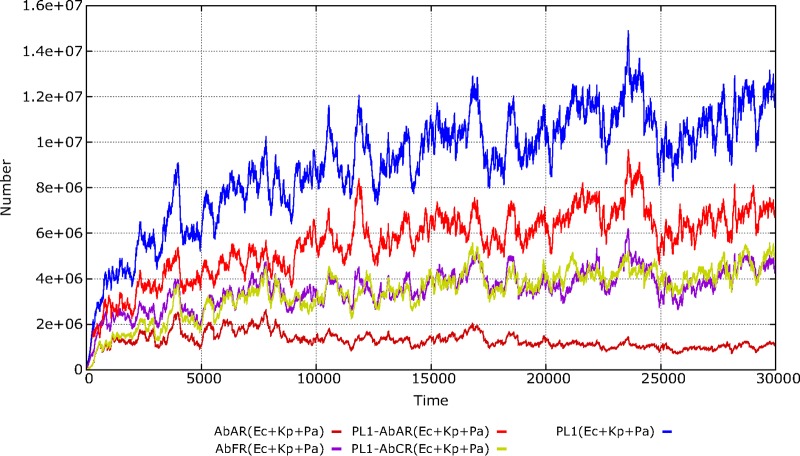
Dynamics of a plasmid and resistance traits in the hospital environment. The species E. coli, K. pneumoniae, and P. aeruginosa are included as a genetic exchange community. In blue, total number of the plasmid PL1; in bright red, plasmid PL1 with the gene AbAR (AMP); in green, PL1 with AbCR (CTX); in violet, chromosomal AbFR (FLQ) gene; in red-brown, chromosomal AbAR (as in K. pneumoniae). In ordinates, number of plasmids or resistance traits in h-cells (packages of 100 identical cells) in all hosts per milliliter (with each host represented by 1 ml of colonic content).

We consider E. coli, K. pneumoniae, and Pseudomonas aeruginosa to be members of a “genetic exchange community” (12, 13) for the plasmid PL1. As shown in Fig. 5, we can compare the evolutionary advantage of the same resistance phenotypic trait (AbAR) harbored in a plasmid, as in E. coli, to that of the trait harbored in the chromosome, as in K. pneumoniae. The overall success of the PL1 plasmid (blue line) benefits from the fact that this mobile element is selected by two different antibiotics (AbA and AbC; resistance shown in red and green lines, respectively). Interestingly, resistance to AbFR (violet) is selected from early stages of the experiment, and after 4,000 steps it converges with the AbCR, a plasmid-mediated trait, meaning that this plasmid is maintained almost exclusively in strains harboring AbFR genes, similarly to empirical findings (14, 15). When the conjugation rate of PL1 was increased, the main effect was the reduction in selection of K. pneumoniae, as the predominance of the PL1-AbAR plasmid from the more abundant populations of E. coli tended to dislodge PL1-AbCR from K. pneumoniae (results not shown).

### Dynamics under conditions of changing scenarios in the hospital and community compartments. (i) Frequency of patient flow between hospital and community.

**FIG 6:**
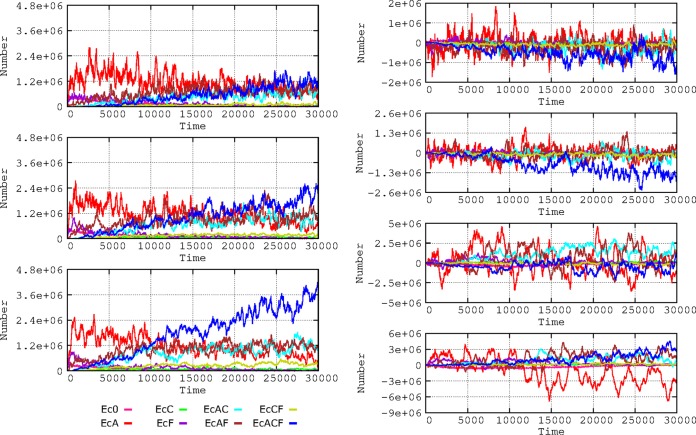
Influence of flow of patients between hospital and community. On the left, influence on E. coli resistance phenotypes in the hospital when one patient is admitted at/discharged from the hospital every 2 (top), 4 (middle), or 8 hours (bottom). On the right, subtractive representation of E. coli phenotypes in the hospital: 2 h versus 4 h (first panel); 4 h vs. 8 h (second panel). Below, E. coli phenotypes in the community with a flow of 2 h versus 4 h (third panel); 4 h vs. 8 h (fourth panel).

The frequency of exchange of individuals between the hospital and the community (hospital admission and discharge rates) influences the evolution of antibiotic resistance (Fig. 6). This occurs because sensitive bacteria enter the hospital with newly admitted patients from the community (where resistance rates are low), and this ‘‘immigration’’ allows sensitive bacteria to ‘‘wash out’’ resistant bacteria (16). Multiresistant E. coli strains emerge much earlier with decreased flow rates, because bacteria resistant to individual drugs have more time to coexist and thus to exchange resistances by gene flow and because the length of “frequent exposure” to different antibiotics (and, consequently, selection) increases (17). The effect of the slow flow of patients to the community is a late reduction in multiresistance (AbAR-AbCR-AbFR) and an earlier reduction in double resistances (AbAR-AbFR and AbAR-AbCR). In the community compartment, however, multiresistance increases when the flow from the hospital is more frequent.

### (ii) Frequency of patients treated with antibiotics.

**FIG 7:**
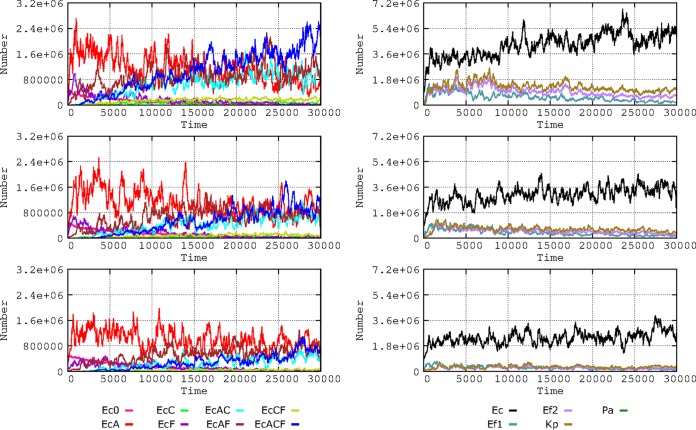
Influence of the frequency of patients treated with antibiotics. On the left, E. coli phenotypes when 20% (top), 10% (middle), or 5% (bottom) of patients received antibiotics during a week at four doses per day. On the right, effect on bacterial species. Colors are as described for Fig. 1 and 2.

Higher proportions of patients exposed to antibiotics increase selection of antibiotic resistance (16). We analyzed this effect in our model, considering proportions of 20%, 10%, and 5% of patients exposed to 7 consecutive days of antibiotic therapy at four doses per day (Fig. 7). If a high proportion (20%) of patients are treated, E. coli multiresistance is efficiently selected, as well as K. pneumoniae and E. faecium resistance. If this proportion is reduced to 10% (and, particularly, to 5%), there is a substantial reduction in the amount of resistant E. coli cells and the emergence of multiresistant bacteria is delayed (individual resistance data not shown for these species). However, the evolution of E. coli toward more multiresistance partially counteracts the selective advantage of these species, restricting their growth to some extent, even under conditions of high densities of treated patients.

### (iii) Frequency of bacterial transmission rates in the hospital.

**FIG 8:**
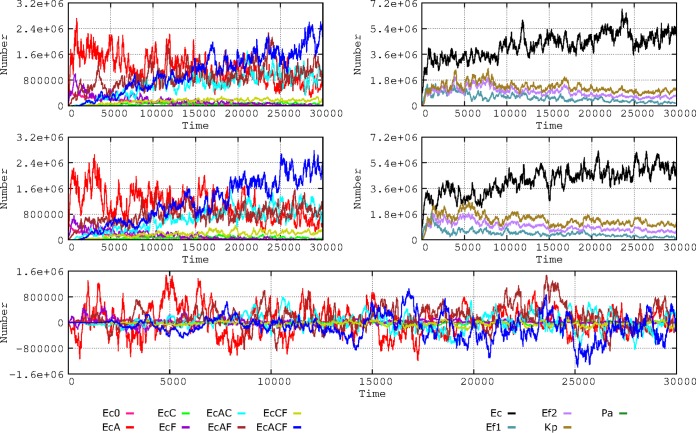
Influence of the frequency of bacterial cross-transmission rates in the hospital. On the left, dynamics of E. coli phenotypes when bacterial exchanges between patients occurred in 5% (top) or 20% (middle) per hour. On the right, influence on the species composition: 5% (top) or 20% (middle). A subtractive representation (5% versus 20%) is provided at the bottom. Colors are as described for Fig. 1 and 2.

Transmission of bacteria (i.e., any type of bacteria, including commensals) among individuals in the hospital influences the spread of antibiotic resistance. The effect of transmission rates of 5% and 20% per hour was analyzed (Fig. 8), and the results expressed the proportion of individuals that acquired any kind of bacteria from another individual per hour. These rates might appear exceedingly high, indicating very frequent transmission between hosts, but we refer here to rates of cross-colonization involving “any type of bacteria.” Normal microbiota transmission rates between hosts have never been measured, such measurements probably requiring a complex metagenomic approach (18). Differences in effects on evolution of E. coli phenotypes in comparisons of 10% and 20% colonization rates are unclear; perhaps 10% transmission produces full effects and 20% does not add much more. The subtractive representation allows discernment of a global advantage for the multiresistant phenotypes (AbAR-AbCR-AbFR) when the proportion of interhost transmission rises from 5% to 20%. The monoresistant AbAR phenotype tends to be maintained longer under conditions of low contagion rates. Note that multiresistant phenotype “bursts” occur (dark blue spikes in the figure) also with low contagion rates (5% box in Fig. 8) and that “bursts” of less-resistant bacteria (red spikes) also occur with high contagion rates (20% box). Notice that the increase in cross-colonization rates favors the transmission not only of resistant populations but also of the more susceptible ones, to a certain extent compensating for the spread of the resistant-phenotype populations.

### (iv) Size of transmitted bacterial load.

The absolute number of intestinal bacteria that are transmitted from one host to another one is certainly a factor influencing the acquisition of resistant (or susceptible) bacteria by the recipient. However, this number is extremely difficult to determine, as it depends not only on the mechanism of transmission (19, 20) but also on the possibility that the recipient might have already harbored bacterial organisms indistinguishable from those that are transmitted (21). On the other hand, efficient transmission able to influence colonic microbiota depends on the number of bacteria in the donor host and on the ability of different bacteria to colonize not only in the lower intestine but also in intermediate locations in the body, probably including the mouth or upper intestine (22). To evaluate the potential effect of different bacterial loads acting as inocula, we considered a final immigrant population reaching the colonic compartment equivalent to 0.1%, 0.5%, and 1% of the donor microbiota. As in previous cases, the evolution of multiresistance favored E. coli (Fig. S4). Multiresistant E. coli emerges earlier and reaches higher levels in higher-count inocula, but less-resistant strains are maintained because the higher-count inocula also contain more susceptible bacteria.

10.1128/mBio.02460-18.4 FIG S4 Influence of the size of the transmitted bacterial load. On the left half of the figure, the data represent E. coli phenotype dynamics in the hospital when the mean transmitted bacterial load was equivalent to 0.1% (up), 0.5% (middle), or 1% (bottom) of the colonic microbiota. On the right side, the data represent evolution of the different species with these transmission loads. Color codes are as described for Fig. 1 and 2. Download FIG S4, EPS file, 3.0 MB. Copyright © 2019 Campos et al. 2019 Campos et al. This content is distributed under the terms of the Creative Commons Attribution 4.0 International license.

### (v) Intensity of the effect of antibiotics on bacterial populations.

**FIG 9:**
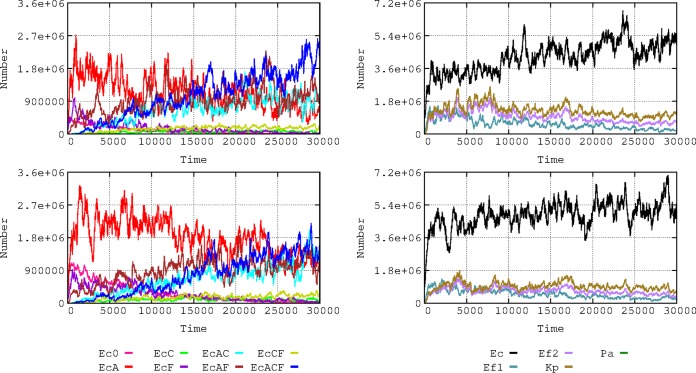
Influence of the activity of the antibiotic on E. coli phenotypes (left) and the species composition (right). (Upper panels) Susceptible bacteria were eliminated at rates of 30% after the first hour of exposure and 15% after the second hour. (Lower panels) The elimination rates were lower: 7.5% after the first hour and 3.75% after the second hour. Colors are as described for Fig. 1 and 2.

The issue of the relationship of the “potency” (intensity of antibacterial activity) of antibiotics to the selection of resistance has been a matter of recent discussions (23–26). To illustrate the point, we changed the bactericidal effect of the antibiotics used in the model. Clinical species were killed at rates of 30% and 15% (reflecting a population decrease) in the first and second hour of exposure, respectively, and these rates were then decreased to 7.5% to 3.75%. Note that these modest killing rates are intended to reflect the diminished effect of antibiotics in slow-growing clinical bacteria located in a complex colonic microbiome. The more susceptible E. coli phenotypes are maintained for longer periods when the killing intensity of antibiotics is lower; in contrast, the multiresistant phenotype emerges earlier and reaches higher numbers when the intensity of antibiotic action increases (Fig. 9). Under conditions of high antibiotic intensity, there is also a (small) increase in the levels of the resistant K. pneumoniae and E. faecium phenotypes. This experiment shows that a high rate of elimination of the more susceptible bacteria favors the colonization by the more resistant ones.

### (vi) Intensity of the antibiotic effect on colonic microbiota.

**FIG 10:**
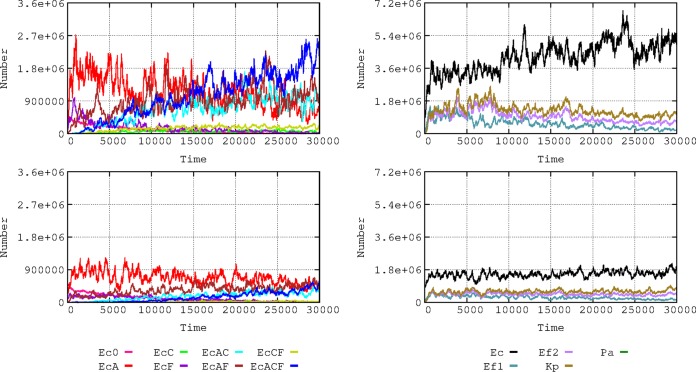
Influence of the intensity of the antibiotic effect on colonic microbiota of patients in the hospital. (Left) Effects on E. coli phenotype of a reduction in microbiota of 25% for AbA, 20% for AbC, and 10% for AbF (top); these values were reduced to 10%, 5%, and 2%, respectively (bottom). (Right) The effects on the species composition.

The proportion of the colonic microbiota killed by antibiotic treatment (and, thus, the size of the open niche for other strains to multiply) constitutes an important factor in the multiplication of potentially pathogenic bacteria and hence affects acquisition (mutational or plasmid-mediated) of resistance and transmission to other hosts. In the basic model, the rates of reduction of the population were 25% for AbA, 20% for AbC, and 10% for AbF; in an alternative scenario, these proportions were modified to 10%, 5%, and 2%, respectively. The results of this change were impressive (Fig. 10): the numbers of bacteria were reduced but also the evolution toward antibiotic resistance (EC) occurred at a lower rate, and even if the proportions of resistance phenotypes were to steadily increase through time, the absolute numbers would not grow, thus limiting host-to-host transmission.

### (vii) Strength of antibiotic selection on resistance traits.

The strength of antibiotic selection is an important parameter in the evolutionary biology of antibiotic resistance (27). Our computational model allows heuristic acquisition of knowledge about the strength of selection of an antibiotic for a particular resistance trait, considering how the resulting trend is (or is not) compatible with the observed reality. An example is the following unanswered question: does plasmid-mediated cefotaxime resistance (AbCR) also provide protection against aminopenicillins (AbAR)? Strains harboring TEM or SHV extended-spectrum beta-lactamases hydrolyzing cefotaxime probably retain sufficient levels of aminopenicillin hydrolysis to be selected by aminopenicillins. However, the cefotaxime-resistant/aminopenicillin-susceptible phenotype is rare in hospital isolates. In our model, this was investigated by providing different strengths of ampicillin (AbA) selection for a cefotaxime-resistant phenotype (AbCR) as follows: no selection (0%), selection in only 10% of the cases (10%), and full selection (100%). The implementation of the model (Fig. S5) showed that if ampicillin were able to select for cefotaxime resistance, the aminopenicillin-susceptible and cefotaxime-resistant phenotype should be prevalent from early stages. This is not what is observed in the natural hospital environment, suggesting that ampicillin is not a major selector for cefotaxime resistance.

10.1128/mBio.02460-18.5 FIG S5 Expected dynamics of hospital-based E. coli under the hypothesis that AbCR might provide the following levels of resistance to AbA: 0% (upper panel), 10% (mid panel), or 100% (lower panel). Color codes are as described for Fig. 1. Download FIG S5, EPS file, 0.1 MB. Copyright © 2019 Campos et al. 2019 Campos et al. This content is distributed under the terms of the Creative Commons Attribution 4.0 International license.

## DISCUSSION

The rate of antibiotic resistance among bacterial species in a given environment is the result of the interaction of biological elements within a framework determined by many local variables, constituting a complex parameter space (28–30). There is a need to consider (in an integrated way) how changes in these parameters might influence the evolution of resistant organisms. This endeavor requires the application of new computational tools that should consider the nested structure of the microbial ecosystems, where mechanisms of resistance (genes) can circulate in mobile genetic elements among bacterial clones and species belonging to genetic exchange communities (12, 13) located in different compartments (as in the hospital or the community). A number of different factors critically influence the evolution of this complex system, such as antibiotic exposure (frequency of treated patients, drug dosages, the strength of antibiotic effects on commensal bacterial communities, and the replication rate of the microbial organisms), as well as the fitness costs imposed by antibiotic resistance, the rate of exchange of colonized hosts between compartments with different levels of antibiotic exposure (hospital and community), or the rates of cross-transmission of bacterial organisms among these compartments. The challenge that we are addressing in this work is that of simultaneously combining for the first time all these factors (and potentially more) in a single computing model to understand the selective and ecological processes leading to the selection and spread of antibiotic resistance. In comparison with the available classic mathematical models that have been applied to the study of evolution of antibiotic resistance (31), the one we are discussing in this work is far more comprehensive in terms of the level of capture of the multilevel parametric complexity of the phenomenon. Note that results obtained with the model and presented here correspond to only a very limited number of possible “computational experiments,” chosen to show the possibilities of the model, but that virtually unlimited numbers of other experiments, with different combinations of parameters, are feasible *à la carte* with a user-friendly interface. In addition, our model can illustrate principles, generate hypotheses, and guide and facilitate the interpretation of empirical studies (32, 33). Examples of these heuristic predictions are that resistance (lower antibiotic effect) in colonic commensal flora can minimize colonization by resistant pathogens, the possible minor role of aminopenicillins in the selection of extended-spectrum beta-lactamases (AbCR), or the possibility of the presence of plasmids conferring aminopenicillin resistance in K. pneumoniae (phenotypically “invisible,” as this organism has chromosomal resistance to the drug).

Our results are presented in terms of the ensemble of biological entities contained in the whole landscape (for instance, in the hospital), aggregated across individual hosts. This “pooling” approach, which originated in ecological studies, has already been used in studies of antibiotic resistance (34). Environments (such as the hospital) are depicted as single “big world” units colonized by “big world populations,” including those that are antibiotic resistant but also the susceptible ones, which can limit the spread of resistance—in a sense, “spreading health” (35). In this scenario, how might antibiotics modify the available colonization space (36, 37)? Our model includes elimination of part of the global colonic microbiota with antibiotic use, favoring the colonization of resistant organisms, which were previously in the minority.

We can reproduce the successive “waves” of increasingly resistant phenotypes in our computational experiments, mimicking the clonal interference phenomenon (38). We show that the speed and intensity of this process depend on the global resistance landscape and the density and phenotype of the bacterial subpopulations. Our model predicts that previous mutational ciprofloxacin resistance facilitates fast evolution of multiresistance by horizontal acquisition of resistance genes (14, 15). We also show that the long-term dissemination of chromosomally encoded genes is far less effective than the spread of traits encoded in transferable plasmids, even though some limitations are detectable because of plasmid incompatibility. A frequently overlooked aspect of antibiotic resistance suggested by the results of our membrane computing experiments is that, over the long term, the evolution of multiresistance probably favors some predominant species such as E. coli, where there is also an increasing benefit for the more resistant clones.

The consequences of changes in the transmission and treatment rates of the hospital and the community were also explored in our model. Several mathematical models have been used to investigate these changes also (16, 37–45). It is clear that reducing discharges and admissions of patient in hospitals has the effect of increasing the local rates of antibiotic resistance, but in our model, increases in the proportions of antibiotic-treated patients in the hospital have a stronger effect, stressing the importance of precision in prescribing antibiotic therapy (44). The increasing rates of hospital cross-colonization also influence the rise of resistance, but this effect seems lower than expected, probably because higher transmission rates also assure transmission of the more susceptible antibiotic populations, in a kind of “washing out” process of resistance, such as that which occurs when the community-hospital flow increases (16). The model also predicts that increases in the “amount” of bacteria transmitted between hosts favor increases of antibiotic resistance. We considered another frequently overlooked factor, namely, the consequences of increases in the “intensity” (aggressiveness) of the antibiotic therapy because of frequent dosage and particularly in terms of its ability to reduce the populations of colonic microbiota and, therefore, the “colonization resistance” for resistant opportunistic pathogens (46).

Precise data are not always easy to obtain, and the type of mathematical or computational models should influence the results of predictions (47). However, because of the functional analogy of membrane computing with the biological world, we hypothesize that the trends revealed in our computational model reflect general processes in the evolutionary biology of antibiotic resistance. If the model were fed with objective data extracted from a real landscape (which would be possible with a user-friendly interface), it could provide a reasonable expectation of the potential evolutionary trends in the particular environment and could support the adoption of corrective interventions (48). Validation of this computational model is the next necessary step; in an approach to this goal, we are developing an “experimental epidemiology” model where the parameters could be altered and measured (49) and are also planning prospective hospital-based observations.

Finally, we stress that the type of membrane computing model that was applied in this work can be easily escalated or adapted to a variety of applications in systems biology (50, 51) and in particular can be used to support efforts to understand complex ecological systems with nested hierarchical structures and involving microorganisms (52).

## MATERIALS AND METHODS

### Software implementation and computing model.

All computational simulations were performed using an updated version of ARES (Antibiotic Resistance Evolution Simulator) (8), which is the software implementation of a P system for the modeling of antibiotic resistance evolution. This P system model works with objects and membranes distributed in different regions organized in a tree-like structure as in the P system classic model but now with more-specific rules: the “object rules” can modify an object (evolution rules) or move the object out, in, or between membranes; and the “membrane rules” can move membranes out, in, or between regions that contain them as “object rules” and can dissolve and duplicate membranes. When a membrane is dissolved, all the membranes and objects inside disappear. For duplication, we can define which objects are to be duplicated and which ones are to be distributed; the membranes are always distributed. The implementation of our P system uses a stochastic method to apply the rules (the rules being ordered by priorities), and each rule has a “probability” to be applied. Other computational objects can be introduced, either to tag particular membranes or to interact with the embedded membranes, for instance, mimicking antibiotics, according to a set of preestablished rules and specifications. We obtain an evolutionary scenario that includes several types of nested computing membranes emulating entities such as (i) resistance genes, located in the plasmid, in other conjugative elements, or in the chromosome; (ii) plasmids and conjugative elements transferring genes between bacterial cells; (iii) bacterial cells; (iv) microbiotas where different bacterial species and subspecies (clones) can meet; (v) hosts containing the microbiotic ensembles; and (vi) the environment(s) where the hosts are contained. The current version of ARES (2.0) can be freely downloaded at https://sourceforge.net/projects/ares-simulator/. ARES 2.0 runs in any computer (is a Java application), albeit it is highly recommendable to install it in at least a 4-by-6-core server with 128 GB of RAM. The original ARES Web site at http://gydb.org/ares offers sections with information about the rules and parameters currently used by ARES.

### Anatomy of the model application.

The current application of the model was structured accordingly with the following composition: (i) compartments containing individual hosts at particular densities, mimicking a hospital (H) and a community environment (C) (flux of individuals between the two compartments occurs at variable rates, mimicking admission or discharge from the hospital); (ii) clinically relevant bacterial populations colonizing these hosts, consisting of the species Escherichia coli, Enterococcus faecium, Klebsiella pneumoniae, and Pseudomonas aeruginosa. These populations diversify from their initial phenotype by acquisition of mutations and/or mobile genetic elements and of PL1 plasmids circulating in E. coli, K. pneumoniae, and P. aeruginosa or of conjugative elements (CO1) in E. faecium. The cell can maintain two copies of the PL1 plasmid (containing resistance to AbA [PL1-AbAR] or AbC [PL1-AbCR]) but not more, so that when a third copy of the PL1 plasmid enters the cell, one of the three is stochastically removed. AbCR produces some degree of resistance to AbA, and we believe that this antibiotic also (in 10% of the cases) selects cells containing plasmid PL1-AbCR. CO1 is an E. faecium “plasmid-like” mechanism of transfer of chromosomal gene AbAR (CO1-AbAR); a single copy of CO1-AbAR exists in the receiving host. Acquisition of (extrinsic) resistance to AbA (AbAR) is mediated by acquisition of PL1 (or CO1), resistance to AbC (AbCR) by acquisition of PL1 containing the AbCR resistance determinant, and resistance to AbF (AbFR) by mutation. Note that the following results occur in our representations: for example, when Ec0 (susceptible) receives PL1 with AbAR, it becomes EcA; when it receives PL1 with AbCR, it becomes Ec2C; and when Ec0, Ec1, and Ec2 mutate to AbFR, they become EcF, EcAF3, and EcCF, respectively. The acquisition of PL1 with AbAR by EcCF or of PL1 with AbCR by EcAF produces the multiresistant strain EcACF.

### Quantitative structure of the basic model application. (i) Hospitalized hosts in the population.

The data corresponding to the number of hosts in the hospital and community environments reflect an optimal proportion of 10 hospital beds per 1,000 individuals in the community (https://data.oecd.org/healtheqt/hospital-beds.htm). In our model, the hospital compartment has 100 occupied beds and corresponds to a population of 10,000 individuals in the community.

The rates of admission and discharge from hospital are equivalent at 3 to 10 individuals/population of 10,000/day (https://www.cdc.gov/nchs/nhds/index.htm). In the basic model, 6 individuals from the community are admitted to the hospital and 6 are discharged from the hospital to the community per day (at approximately 4-h intervals). Patients are stochastically admitted or discharged, meaning that about 75% of the patients stay in the hospital between 6 and 9 days.

The bacterial colonization space of the populations of the clinical species considered here (Table 1) and of other basic colonic microbiota populations is defined as the volume occupied by these bacterial populations. Under natural conditions, the sum of these populations is estimated in 10^8^ cells per ml of the colonic content. Clinical species constitute only 1% of the cells in each milliliter and have a basal colonization space of 1% of each milliliter of colonic content (0.01 ml). How these spaces are considered for counting populations in the model is explained in the next section.

The ensemble of other populations of microbiota is considered in our basic study model as an ensemble surrounded by a single membrane. The colonic space occupied by these populations can change because of antibiotic exposure. Throughout a course of treatment (7 days), the antibiotics AbA, AbC, and AbF reduce the intestinal microbiota 25%, 20%, and 10%, respectively. As an example, if we consider that 10% of the basic colonic populations was eliminated by antibiotic exposure, their now empty space (0.1 ml) would be occupied by antibiotic-resistant clinical populations and by the colonic populations that survived the challenge. In the absence of antibiotic exposure, the colonic populations are restored to their original population size in two months. Clinical populations are comparatively faster in colonizing the empty space.

### (ii) Populations’ operative packages and counts.

To facilitate the process of model running, we consider that a population of 10^8^ cells in nature is equivalent to 10^6^ cells in the model. In other words, one “hecto-cell” (h-cell) in the model represents an “operative package” of 100 cells in the real world. Because of the very high effective population sizes in bacteria, these 100 cells are considered representative of a uniform population of a single cell type. A certain increase in stochasticity might occur because of using h-cells; however, run replicates do not differ significantly (see Fig. S1 in the supplemental material). Also, for computational efficiency, we considered that each patient (in a hospital) or individual (in the community compartment) is represented in the model by 1 ml of its colonized colonic space (about 3,000 ml) and refer to the corresponding value as a “host-ml.” Consequently, in most of the figures we represent our results as a numbers of h-cells in all hosts per milliliter.

### (iii) Quantitative distribution of clinical species and clones.

In the basal scenario, the distribution of species in these 1,000,000 cells (contained in 1 ml) is as follows: for E. coli, 860,000 cells, including 500,000 susceptible cells, 250,000 cells containing PL1-AbAR, 100,000 cells with the AbFR mutation, and 10,000 cells with both PL1-AbAR and the AbFR mutation; for E. faecium, 99,500 AbA susceptible and 20,000 AbAR; for K. pneumoniae, 20,000 with chromosomal AbAR, PL1-AbCR, and AbFR; for P. aeruginosa, 500 containing PL1-AbCR. At time zero, the distributions are identical in hospitalized and community patients.

### (iv) Tagging starting clone populations in E. coli.

To be able to follow the evolution of particular lineages inside E. coli, four ancestral clones (Ecc) were distinguished, differing in the original resistance phenotype (with Ecc0 as a fully susceptible clone, EccA harboring PL1 determining AbAR, EccF harboring AbFR, and EccAF with PL1-AbAR and AbFR) (Table 1). Each of these clones is tagged at time zero with a distinctive “object” in the model which remains fixed to the membrane, multiplies with the membrane, and is never lost. Each of the daughter membranes throughout the progeny can alter its phenotype by mutation or lateral gene acquisition, but the ancestral clone remains detectable.

### (v) Multiplication rates.

We consider the basal multiplication rate (of 1) the rate corresponding to Ec0, where each bacterial cell gives rise to two daughter cells every hour. In comparison, the rate for E. faecium is 0.85, that for K. pneumoniae is 0.9, and that for P. aeruginosa is 0.15. The acquisition of a mutation, a plasmid, or a mobile element imposes an extra cost corresponding to a value of 0.03. Therefore, the rate for Ec0 is 1, that for EcA is 0.97 (because of the cost of PL1-AbAR), that for EcC is 0.97 (cost of PL1-AbCR), that for EcF is 0.97 (cost of mutation), that for EcAF is 0.94 (PL1-AbAR and AbFR), that for Ef(1) is 0.85, that for Ef(2) is 0.79 (CO1-AbAR and AbFR), that for K. pneumoniae is 0.84 (PL1-AbAR and AbFR), and that for P. aeruginosa with PL1-AbCR is 0.12 (PL1-AbCR). The number of cell replications is limited according to the available space (see above).

Transfer of bacterial organisms from one host to another is expressed by the proportion of individuals that can stochastically produce an effective transfer of commensal or clinical bacteria or susceptible or resistant bacteria to another individual (contagion index [CI]). If contagion is 5% (i.e., if the CI = 5), that means that among 100 patients, 5 “donors” transmit bacteria to 5 “recipients” per hour. In the case of the basic scenario, CI = 5 in the hospital and CI = 1 in the community (all data corresponding to results with CI = 0.01 are available on request). In the basic scenario, donors contribute to the colonic microbiota of recipient individuals with 0.1%, 0.5%, and 1% of their own bacteria. These inocula do not necessarily reflect the number of cells transferred but do reflect endogenous multiplication after transfer, as proposed in other models (53). In any case, cross-transmission is responsible for most new acquisitions of pathogenic bacteria (54).

The frequency of plasmid transfer between bacteria occurs randomly and reciprocally at equivalently high rates among E. coli and K. pneumoniae populations; in the basic model, the rate is 0.0001, representing one effective transfer occurring in 1 of 10,000 potential recipient cells. Plasmid transfer occurs at a lower rate of 0.000000001 in the interactions of E. coli and K. pneumoniae with P. aeruginosa. Conjugative element-mediated transfer of resistance among E. faecium populations occurs at a frequency of 0.0001, but E. faecium bacteria are unable to receive resistance genes from or donate resistance genes to any of the other bacteria considered. In the case of E. coli and K. pneumoniae plasmids, we consider plasmid limitation in the number of accepted plasmids such that if a bacterial cell with two plasmids receives a third plasmid, there is a stochastic loss of one of the residents or the incoming plasmid but all three cannot coexist in the same cell.

Mutational resistance is considered only in the present version of the model for resistance to AbF (fluoroquinolones). Organisms of the model-targeted populations mutate to AbF at the same rate, i.e., 1 mutant every 10^8^ bacterial cells per cell division.

### (vi) Antibiotic exposure.

In the basic model, 5%, 10%, or 20% of the individuals in the hospital compartment are exposed to antibiotics each day, each individual being exposed (treated) for 7 days. In the community compartment, 1.3% of individuals are receiving treatment, with each of them also exposed to antibiotics for 7 days. Antibiotics AbA, AbC, and AbF are used in the hospital and the community compartments at proportions of 30%, 40%, and 30% and of 75%, 5%, and 20%, respectively. In the basic scenario, a single patient is treated with only one antibiotic that is administered every 6 h.

### (vii) Intensity of the effect of antibiotics on susceptible clinical populations.

After each dose is administered, all three (bactericidal) antibiotics induce a decrease of 30% in the susceptible population after the first hour of dose exposure and 15% in the second hour. These relatively modest bactericidal effects reflect the reduction in the antibiotic killing rates of clinical populations inserted into the colonic microbiota. The antibiotic stochastically penetrates at those percentages of bacterial cells, and those that are susceptible are removed (killed). Therapy is maintained in the treated individual for 7 days.

### (viii) Intensity of the effect of antibiotics on colonic microbiota.

Antibiotics exert an effect that reduces the density of the colonic commensal microbiota, resulting in free space and nutrients that can benefit the clinical populations. In the basic model, the levels of such reductions are 25% for AbA, 20% for AbC, and 10% for AbF.
